# Precision medicine for long-term depression outcomes using the Personalized Advantage Index approach: cognitive therapy or interpersonal psychotherapy?

**DOI:** 10.1017/S0033291719003192

**Published:** 2021-01

**Authors:** Suzanne C. van Bronswijk, Robert J. DeRubeis, Lotte H. J. M. Lemmens, Frenk P. M. L. Peeters, John R. Keefe, Zachary D. Cohen, Marcus J. H. Huibers

**Affiliations:** 1 Department of Clinical Psychological Science, Maastricht University, Maastricht, The Netherlands; 2 Department of Psychology, University of Pennsylvania, Philadelphia, USA; 3 Department of Psychiatry, Weill Cornell Medical College, New York, USA; 4 Department of Psychiatry, University of California, Los Angeles, Los Angeles, CA, 90095, USA; 5 Department of Clinical Psychology, VU University Amsterdam, Amsterdam, The Netherlands

**Keywords:** Cognitive therapy, depression, interpersonal psychotherapy, precision medicine, prediction

## Abstract

**Background:**

Psychotherapies for depression are equally effective on average, but individual responses vary widely. Outcomes can be improved by optimizing treatment selection using multivariate prediction models. A promising approach is the Personalized Advantage Index (PAI) that predicts the optimal treatment for a given individual and the magnitude of the advantage. The current study aimed to extend the PAI to long-term depression outcomes after acute-phase psychotherapy.

**Methods:**

Data come from a randomized trial comparing cognitive therapy (CT, *n* = 76) and interpersonal psychotherapy (IPT, *n* = 75) for major depressive disorder (MDD). Primary outcome was depression severity, as assessed by the BDI-II, during 17-month follow-up. First, predictors and moderators were selected from 38 pre-treatment variables using a two-step machine learning approach. Second, predictors and moderators were combined into a final model, from which PAI predictions were computed with cross-validation. Long-term PAI predictions were then compared to actual follow-up outcomes and post-treatment PAI predictions.

**Results:**

One predictor (parental alcohol abuse) and two moderators (recent life events; childhood maltreatment) were identified. Individuals assigned to their PAI-indicated treatment had lower follow-up depression severity compared to those assigned to their PAI-non-indicated treatment. This difference was significant in two subsets of the overall sample: those whose PAI score was in the upper 60%, and those whose PAI indicated CT, irrespective of magnitude. Long-term predictions did not overlap substantially with predictions for acute benefit.

**Conclusions:**

If replicated, long-term PAI predictions could enhance precision medicine by selecting the optimal treatment for a given depressed individual over the long term.

## Introduction

Optimizing treatment selection is a promising approach to improve psychotherapy outcomes for major depressive disorder (MDD, Cohen and DeRubeis, 2018). Although research shows that different types of psychotherapy for MDD are equally effective on average (Cuijpers *et al*., 2011), an individual's response to different therapies may vary greatly (Simon and Perlis, 2010). In addition, treatment response is highly unpredictable; for example, individuals often go through multiple antidepressant therapies before an effective regimen is identified (Rush *et al*., 2006). Treatment selection aims to move beyond average effectiveness and focuses on the question, ‘What works for whom?’ Efforts to match individuals with specific treatments are referred to as personalized or precision medicine (Simon and Perlis, 2010; Katsnelson, 2013; Cohen and DeRubeis, 2018).

To optimize treatment selection, individual characteristics that reliably predict differential treatment outcomes, the so-called moderators or prescriptive variables, need to be identified. Biomarkers (e.g. genetic or brain imaging variables), clinical features (e.g. illness severity or chronicity), and sociodemographic characteristic (e.g. gender or education level) have been the focus of efforts to identify useful moderators. However, no single moderator is likely to be robust enough, on its own, to reliably guide treatment selection in MDD (Simon and Pris, 2010; Cohen and DeRubeis, 2018; Kessler, 2018), and indeed none have been identified. In recent years, the development of multivariate prediction models, which aggregate multiple moderators, has shown promise as a means of producing powerful predictions (Cohen and DeRubeis, 2018). These models aim to convert the predictive information of multiple moderators into actionable recommendations to guide treatment selection. Examples of these multivariate models are the ‘matching factor’ (Barber and Muenz, 1996), the ‘nearest-neighbors’ approach (Lutz *et al*., 2006), and the M* approach (Kraemer, 2013; Wallace *et al*., 2013; Smagula *et al*., 2016; Niles *et al*., 2017*a*, 2017*b*).

Another promising multivariate approach to guide treatment selection between two or more treatments is the Personalized Advantage Index (PAI, DeRubeis *et al*., 2014). This method not only provides an individual treatment recommendation, it also delivers a quantitative estimate of the predicted advantage of the indicated treatment over the non-indicated treatment(s). These recommendations are based on the difference between predicted outcomes of two or more treatments using a model that includes multiple predictors and moderators. DeRubeis *et al*. (2014) developed and introduced this approach by predicting outcomes of acute-phase cognitive therapy (CT) and pharmacotherapy. Since then, the PAI approach has been replicated and extended to acute phase CT *v.* interpersonal psychotherapy (IPT) for MDD (Huibers *et al*., 2015), continuation CT *v.* fluoxetine for recurrent MDD (Vittengl *et al*., 2017), sertraline *v.* placebo for MDD (Webb *et al*., 2019), trauma-focused cognitive behavioral therapy (CBT) and eye movement desensitization for posttraumatic stress disorder (PTSD) (Deisenhofer *et al*., 2018), and dropout in MDD (Zilcha-Mano *et al*., 2016) and PTSD (Keefe *et al*., 2018).

In the current study, we aim to extend the PAI approach for treatment selection to focus on longer-term depression outcomes within the context of a 17-month follow-up of a recent randomized trial comparing CT and IPT (Lemmens *et al*., 2015, 2019). CT and IPT are two frequently practiced psychotherapies for MDD and have been shown to be equally effective in the acute phase (Jakobsen *et al*., 2012; Lemmens *et al*., 2015) with comparable prophylactic effects after treatment termination (Lemmens *et al*., 2019). The current study extends a recently published PAI effort predicting acute treatment response (post-treatment point estimates) using a completer's subset of the same study sample (the ‘post-treatment’ PAI, Huibers *et al*., 2015; Lemmens *et al*., 2015). In the current study, a ‘long-term’ PAI was built. First, we selected pre-treatment variables using a two-step *machine learning approach*, to identify reliable predictors and moderators of long-term depression outcome after CT and IPT. Second, we calculated PAI scores for individual treatment recommendations based on a final model that combined the selected predictors and moderators with a cross-validation approach. The utility of the long-term PAI recommendations was then evaluated by comparing the set of predictions with the respective observed follow-up outcomes. In addition, the long-term PAI scores per individual were compared with the post-treatment PAI scores to examine if the PAI scores for that individual overlap, and if the different intended outcomes (optimal post-treatment outcomes *v.* optimal long-term outcomes) led to different treatment recommendations. Finally, a secondary analysis was conducted, repeating the process of variable selection and model fitting to a fivefold held-out sample (instead of the full sample) to create five separate models. The predictions of these models were then compared to the long-term PAI predictions, to provide an insight into the method's robustness (e.g. the risk of overfitting), and its potential for out-of-sample predictions.

## Methods

### Design and participants

Data come from a randomized controlled trial into the effectiveness of individual CT and IPT for MDD. Adult outpatients (18–65 years) were recruited from the mood disorders unit of the Academic Maastricht Outpatient Mental Health Centre (RIAGG Maastricht, the Netherlands). Inclusion criteria were a primary diagnosis of MDD (confirmed with the Structured Clinical Interview for DSM-IV Axis I disorders; First *et al*., 1995), internet access, an email address, and sufficient knowledge of the Dutch language. Individuals with bipolar- or highly chronic depression (current episode >5 years) were excluded from the study. Other exclusion criteria were a high acute suicide risk, concomitant pharmacological or psychological treatment, drugs and alcohol abuse/dependence, and an IQ lower than 80. After providing written informed consent, a total of 182 participants were randomly assigned to CT (*n* = 76), IPT (*n* = 75), or a 2-month waiting-list control (*n* = 31) followed by treatment of choice. For the current study, we limited our sample to the two active conditions (*n* = 151) and included pre-treatment variables and outcome data from the follow-up phase (month 7–24).

### Treatments

Treatment consisted of 16–20 individual 45-min sessions (M = 17, s.d. = 2.9) that were planned weekly and were allowed to be less frequent toward the end of therapy. CT was carried out following the guidelines by Beck *et al*. (1979). IPT was based on the manual by Klerman *et al*. (1984). Therapists were 10 licensed psychologists, psychotherapists, and psychiatrists with substantial clinical experience (M = 9.1 years, s.d. = 5.4). For both CT and IPT, treatment quality was rated by independent assessors as ‘(very) good’ to ‘excellent’ (Lemmens *et al*., 2015). During follow-up, individuals were free to seek additional treatment for MDD, including psychological support (*n* = 54, one or more sessions with a general practitioner or a mental health care professional) and antidepressant medication (*n* = 29).

### Measures

#### Primary outcome

Primary outcome was depression severity measured with the Beck Depression Inventory, second edition (BDI-II, Beck *et al*., 1996) during follow-up at 7, 8, 9, 10, 11, 12, and 24 months. These BDI-II scores were aggregated, for each participant, into an Area under the Curve (AUC) to obtain an overall measure of depression severity across the 17-month follow-up period. The AUC can be interpreted as a summary of depressive symptom burden measured over several time points.

#### Pre-treatment variables

We examined 69 pre-treatment variables from six previously described domains: (1) depression variables, (2) demographics, (3) psychological distress, (4) general functioning, (5) psychological processes, and (6) life and family history (Fournier *et al*., 2009; Huibers *et al*., 2015). A correlation matrix corrected for attenuation was computed for all 69 variables. Variables that were highly correlated (cor. > 0.70) with other variables were removed to prevent multicollinearity. Choices on which one of two variables should be removed depended on redundancy (e.g. multiple indicators for quality of life) and interpretability (e.g. including a total scale instead of highly correlated subscales of one measurement instrument) and were always made as a group decision of the research team. Similar pre-selection procedures have been described in previous studies (Lorenzo-Luaces *et al*., 2017; Kim *et al*., 2019). As a result of this procedure, we removed 31 variables, and the remaining 38 pre-treatment variables were selected for further analyses (see Table 1). They came from the following measurement scales: Beck Hopelessness Scale (BHS, Beck and Steer, 1988), Brief Symptom Inventory (BSI, Derogatis and Melisaratos, 1983), Structured Clinical Interview for DSM-IV Axis I disorders (SCID-I, First *et al*., 1995), Structured Clinical Interview for DSM-IV Axis II disorders (SCID-II, First *et al*., 1997), Work and Social Adjustment Scale (WSAS, Mundt *et al*., 2002), Dysfunctional Attitudes Scale (DAS, Weissman and Beck, 1978; de Graaf *et al*., 2009), Inventory of Interpersonal Problems (IIP, Horowitz *et al*., 1988), Self-Liking and Self-Competence Scale Revised (SLSC, Tafarodi and Swann, 2001; Vandromme *et al*., 2007), Ruminative Response Scale (RRS, Raes *et al*., 2003), and Attributional Style Questionnaire (ASQ, Peterson *et al*., 1982; Cohen *et al*., 1986).

**Table 1.** Description and comparison of pre-treatment variables in cognitive therapy *v.* interpersonal psychotherapy

	Cognitive therapy (*n* = 76)	Interpersonal psychotherapy (*n* = 75)
DOMAIN I: Depression		
Recurrent depressive episodes, yes, *n* (%)	38 (50.0)	36 (48.0)
Hopelessness, BHS, mean (s.d.)	11.3 (4.9)	12.4 (4.7)
DOMAIN II: Demographics		
Female sex, *n* (%)	54 (71.1)	46 (61.3)
Age, mean (s.d.)	41.2 (12.4)	41.3 (11.8)
Partner, yes, *n* (%)	43 (56.6)	51 (68.0)
Education level		
Low, *n* (%)	16 (21.0)	13 (17.3)
Intermediate, *n* (%)	48 (63.2)	41 (54.7)
High, *n* (%)	12 (15.8)	21 (28.0)
Active employment, yes, *n* (%)	43 (56.6)	47 (62.7)
Treatment expectancy, mean (s.d.)	6.8 (1.2)	6.5 (1.3)
DOMAIN III: Psychological distress		
General psychological distress, BSI		
Somatic complaints, mean (s.d.)	7.8 (5.7)	7.7 (4.7)
Cognitive problems, mean (s.d.)	11.0 (5.0)	11.6 (5.0)
Depression, mean (s.d.)	10.7 (4.7)	12.1 (5.1)
Anxiety, mean (s.d.)	7.7 (5.0)	8.8 (4.6)
Phobic Anxiety, mean (s.d.)	4.8 (4.0)	4.8 (3.9)
Hostility, mean (s.d.)	4.4 (3.3)	5.1 (3.9)
Paranoid Symptoms, mean (s.d.)	6.6 (4.4)	7.2 (4.1)
Number of comorbid axis I disorders, SCID-I, mean (s.d.)	0.6 (0.8)	0.7 (0.7)
Number of comorbid axis II disorders, SCID-II, mean (s.d.)	0.7 (1.0)*	0.4 (0.7)*
Number of comorbid axis II traits, SCID-II, mean (s.d.)	0.3 (0.7)*	0.6 (1.0)*
DOMAIN IV: General functioning		
Social and work functioning, WSAS, mean (s.d.)	23.2 (7.7)	22.4 (7.2)
Level of impairment, RAND-36		
Physical functioning, mean (s.d.)	73.7 (23.0)	74.1 (20.5)
Social functioning, mean (s.d.)	41.5 (19.2)	40.8 (20.2)
Role limitations (physical problems), mean (s.d.)	37.0 (41.0)	33.7 (38.4)
Role limitations (emotional problems), mean (s.d.)	17.8 (32.1)	13.3 (25.1)
General health perception, mean (s.d.)	46.7 (16.5)	43.8 (14.1)
Perceived health change during past year, mean (s.d.)	31.3 (26.0)	26.0 (24.8)
DOMAIN V: Psychological processes		
Dysfunctional beliefs, DAS		
Factor 1, mean (s.d.)	35.7 (11.1)	36.8 (11.5)
Factor 2, mean (s.d.)	25.4 (6.4)	26.1 (7.1)
Interpersonal problems, IIP, mean (s.d.)	83.1 (24.7)	89.7 (33.9)
Self Liking and Self Competence, SLSC-R, mean (s.d.)	39.3 (8.6)	37.6 (10.7)
Rumination, RRS, mean (s.d.)	49.1 (9.1)	52.4 (8.4)
Attributional Style, ASQ, mean (s.d.)	0.03 (0.9)	0.02 (1.2)
DOMAIN VI: Life and family history		
Number of life events past year, mean (s.d.)	1.8 (1.4)	1.8 (1.3)
Number of childhood trauma events, mean (s.d.)	0.9 (1.3)	0.8 (1.2)
Parental: one or both parents		
In treatment for illness, *n* (%)	19 (25.0)	17 (22.7)
With an anxiety disorder, *n* (%)	13 (17.1)	14 (18.7)
With depression, *n* (%)	35 (46.1)	30 (40.0)
With alcohol abuse, *n* (%)	9 (11.8)	13 (17.3)
With suicidality, *n* (%)	6 (7.9)	11 (14.7)

BHS, Beck Hopelessness Scale; Treatment expectancy, 0 = not successful 10 = very successful; BSI, Brief Symptom Inventory; SCID-I, Structured Clinical Interview for DSM-IV Axis I disorders; SCID-II, Structured Clinical Interview for DSM-IV Axis II disorders; WSAS, Work and Social Adjustment Scale; DAS, Dysfunctional Attitudes Scale; IIP, Inventory of Interpersonal Problems; SLSC-R, Self Liking and Self Competence Scale Revised; RRS, Ruminative Response Scale; ASQ, Attributional Style Questionnaire.

* *p* < 0.05.

### Statistical analyses

#### Variable description and missing data

Between treatment differences of the 38 variables were examined, using *t* tests and χ^2^ tests where appropriate. Missing BDI-II outcomes and variables were imputed using a non-parametric random forest approach (R package ‘MissForest’, Stekhoven and Bühlmann, 2012). This imputation approach has been shown to be accurate and comparable to multiple imputation, with lower imputation errors compared to many other imputation methods (Stekhoven and Bühlmann., 2012; Waljee *et al*., 2013). For the imputation model, we used the following information as input: (1) change scores from baseline of all non-missing BDI-II outcomes (at 3, 7, 8, 9, 10, 11, 12, and 24 months); (2) all scores on non-missing variables; (3) change scores from baseline to post-treatment of all non-missing variables; (4) the received treatment (CT/IPT). To test the imputation method, it was applied to the complete (non-missing) dataset with artificially produced missing data. Imputed values were then compared with actual data values by estimating the normalized root mean squared error (NRMSE) for continuous data and the proportion of falsely classified entries (PFC) for categorical data (Stekhoven and Bühlmann, 2012).

#### Outcome transformation

To produce estimates of ‘overall’ depression severity across the 17-month follow-up phase, BDI-II scores at 7, 8, 9, 10, 11, 12, and 24 months were combined into an AUC using cubic splines to compute integrals. As described elsewhere (Lemmens *et al*., 2015), BDI-II scores between CT and IPT differed at baseline, though the difference was a non-significant trend. To adjust for this difference, we calculated the residuals of a regression function with the AUC as the dependent variable and the BDI-II at baseline as the independent variable. We used these residuals as the outcome variable for further analyses. To avoid confusion, we will refer to these residuals as the AUC.

#### Variable transformation

Discrete and categorical variables were centered, and continuous variables were standardized. Discrete variables with a non-normal distribution were transformed using a log transformation or a square root transformation based on visual inspection (details about transformations can be found in Supplementary Methods I).

#### Variable selection

We used a two-step machine learning approach to select predictors and moderators of long-term outcome in CT and IPT, which has been employed previously (Zilcha-Mano *et al*., 2016; Keefe *et al*., 2018). First, we applied a model-based recursive partitioning method using a random forest algorithm (R package ‘mobForest’, Garge *et al*., 2013). This method splits bootstrapped samples repeatedly into two subgroups based on a pre-determined model. In the current analyses, this pre-determined model was a regression model with the AUC as the dependent variable and the pre-treatment variables as interactions with treatment (*y* = *x* × treatment) to test their potential as moderators. At each potential split, a random subset of variables was available to inform the split, and the data were divided on the variable with the strongest moderator impact, to produce a tree-like structure. By repeatedly using different random subsets of variables, variables with smaller effects were less likely to be dominated by the presence of stronger variables (Strobl *et al*., 2008). Parameters were set as follows: a total of 10 000 trees were computed with a minimum *α* level of 0.10 for splitting and a minimum subgroup size for splits of 15 individuals. As an output of this method, variables were ranked based on a variable importance score indicating their predictive impact. The variable importance score was computed by subtracting the predictive accuracy of a variable when applying the real values, from the predictive accuracy of a variable when applying randomly permuted values. The higher the difference between the real and permutated values, the higher the variable importance. Variables were selected for the second step if they exceeded the threshold, which is the absolute value of the variable importance score of the lowest ranking variable. The second step involves a backward elimination approach using multiple bootstrapped samples (R package ‘bootstepAIC’, Austin and Tu, 2004; Rizopoulos and Rizopoulos, 2009). For this approach, a regression model was specified with the AUC as the dependent variable and the variables selected in the first variable selection step as independent variables, along with their interactions with treatment. A total of 1000 bootstrapped samples of the original data was generated, and backwards elimination (using *α* = 0.05) with the specified model was applied to each of these samples. For each variable, the number of times it was selected and had a positive or negative regression coefficient was computed. If variables were selected in at least 60% of the bootstrapped samples, they were considered robust (Austin and Tu, 2004) and used to build the PAI. For the final moderators, the Johnson–Neyman technique was applied to examine at which value the between treatment difference was significant (Johnson and Neyman, 1936).

#### Building the PAI

The PAI method was applied to generate personalized treatment recommendations based on pre-treatment predictors and moderators (DeRubeis *et al*., 2014). For this approach, the selected variables were combined into a regression model with the AUC as the dependent variable. The independent variables were the predictors, the moderators interacting with the treatment, and the main effects of the moderators. Based on this regression model, individual outcome predictions for each treatment were made using a fivefold cross-validation. With the fivefold cross-validation, the sample was split into five equal groups and individual outcomes of each group were predicted using the regression model with weights based on the data of the other four groups of the sample (the ‘training dataset’, Picard and Cook, 1984). Applying the cross-validation approach reduces the risk of overfitting by not including the individual's data during the computation of regression parameters. For each individual, two separate predictions were made: one predicted score for the treatment the individual actually received (factual) and one predicted score for the treatment the individual did not receive (counterfactual). The differences between these two predictions resulted in a positive or negative score indicating the optimal treatment: a PAI indicating CT or IPT. In addition, the magnitude of this score indicated the strength of the predicted advantage of the indicated PAI treatment, with higher scores representing a stronger need for a specific treatment.

#### Evaluating the PAI

To test the utility of the PAI, actual follow-up outcomes (AUCs) of individuals receiving the PAI-indicated treatment were compared with those of individuals receiving the PAI non-indicated treatment, using *t* tests. Following DeRubeis *et al*. (2014), we also compared the observed follow-up outcomes (AUCs) of those with the highest 60% (absolute values) PAI scores. After that, we evaluated the PAI effect separately for CT and IPT. For participants whose PAI indicated CT, we compared the actual follow-up outcomes (AUCs) of those who received CT (indicated) *v.* those who received IPT (not-indicated). Likewise, for participants whose PAI indicated IPT, we compared actual follow-up outcomes (AUCs) of those who received IPT with those who received CT. We repeated these PAI-indicated CT and IPT comparisons in the subset of participants with the highest 60% of the PAI scores. Finally, we compared the long-term PAI score with the previously reported post-treatment PAI score for each individual, by comparing treatment recommendations (χ^2^ test) and the magnitude of the predicted advantage (correlations). Since a completer subset of the study sample was used to build the post-treatment PAI, we limited this comparison to this smaller subset of individuals (*n* = 134, Huibers *et al*., 2015). For all comparisons, the follow-up AUCs were converted to ‘average follow-up BDI-II scores’ across the 17-month period by dividing the AUC by time in months. Since the AUC and the ‘average BDI-II score’ are interchangeable, we choose to use the latter one (labeled as ‘follow-up BDI-II scores/follow-up depression severity’) for the remainder of this paper, to enhance interpretation and readability of the results.

#### Testing robustness of variable selection and model fitting

For the two-step machine learning approach and model fitting, we used the full sample. Although we applied a cross-validation method to compute the PAI scores, it is still possible that they may be inflated due to double-dipping (i.e. performing variable selection and model fitting in the same sample, Vul *et al*., 2009; Fiedler, 2011). To examine if this affected the results, we ran secondary analyses repeating the process of variable selection and model fitting to a fivefold held-out sample creating five separate models. The predictions of these models were compared with the actual follow-up outcomes. These evaluations were then compared with the evaluations of the main method. Comparisons of these evaluations indicated the potential influence of overfitting, the method's robustness and the potential for out-of-sample predictions.

## Results

### Variable description and missing data

Table 1 presents the differences between treatment groups on the 38 pre-treatment variables. On average, participants who received CT had a higher number of comorbid axis II disorders and a lower number of axis II traits as compared to IPT (*t* = 2.00, df = 144, *p* = 0.047 and *t* = 2.31, df = 144, *p* = 0.02 for disorders and traits, respectively). The other pre-treatment variables did not differ significantly between CT and IPT. A total of 25 observations of all 38 variables were missing (0.4%). On the BDI-II (7, 8, 9, 10, 11, 12, and 24 months), 164 values were missing (15.5%). Of all participants, 139 individuals (92.1%) had no missing variables and 119 individuals (78.1%) had no missing BDI-II scores. Imputation was proven to be accurate when applied to the complete (non-missing) data with artificially produced missing data; the estimated NRMSE was 0.09 and the estimated PFC was 0.02.

### Variable selection

**Fig. 1.**
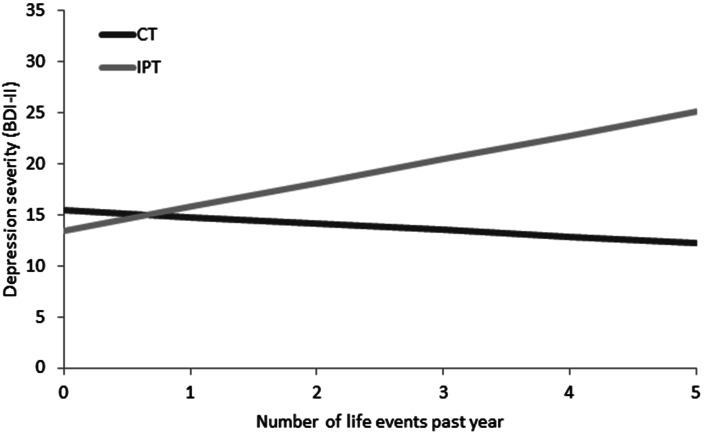
Regression-based estimated means of the average follow-up BDI-II scores (measured at 7, 8, 9, 10, 11, 12, and 24 months) as a function of a number of life events. *Note*: These estimates are based on the final regression model with the other model values set to sample mean. Sample description: 0 life events (*n* = 33), 1 life event (*n* = 38), 2 life events (*n* = 32), 3 life events (*n* = 32), 4 life events (*n* = 11), 5 life events (*n* = 4), 6 life events (*n* = 1). BDI-II, Beck Depression Inventory, Second Edition.

**Fig. 2.**
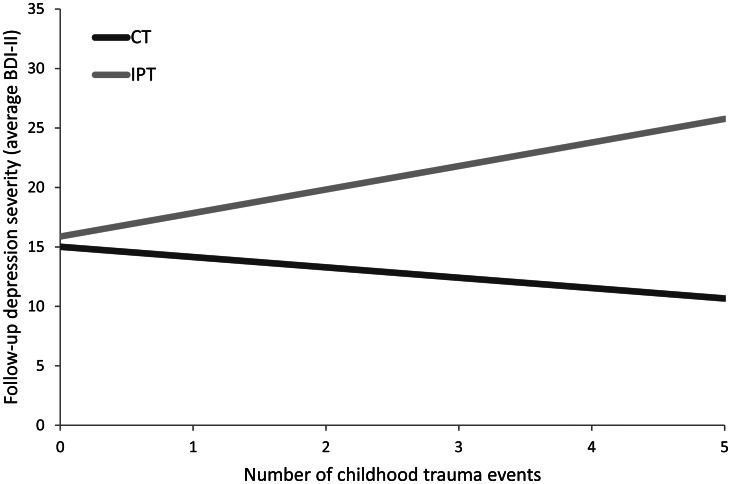
Regression-based estimated means of the average follow-up BDI-II scores as a function of a number of childhood trauma events. *Note*: These estimates are based on the final regression model with the other model values set to sample mean. Sample description: 0 childhood trauma events (*n* = 84), 1 childhood trauma events (*n* = 33), 2 childhood trauma events (*n* = 15), 3 childhood trauma events (*n* = 12), 4 childhood trauma events (*n* = 4), 5 childhood trauma events (*n* = 3). BDI-II, Beck Depression Inventory, Second Edition

The model-based recursive partitioning technique selected the following four variables (ranked from higher to lower variable importance): number of life events in the past year, number of traumatic events in childhood, score on the SLSC-R (a measure of self-esteem), and parental alcohol abuse (yes/no). Of these variables, three variables were selected in at least 60% of the bootstrapped samples using the backwards elimination technique: parental alcohol abuse was identified as a predictor and number of life events past year and number of childhood trauma events were selected as moderators. For parental alcohol abuse, the regression coefficients across the bootstrapped samples were stable with a positive value in 99.8% of the samples indicating that a history of parental alcohol abuse was associated with higher BDI-II scores during the 17-month follow-up phase. As illustrated in Fig. 1, individuals with more recent life events were more likely to have lower overall follow-up BDI-II scores in CT as compared to IPT. Results of the Johnson–Neyman technique indicated that this between-treatment difference was significant for individuals with two or more life events. In Fig. 2, the moderator effect of childhood trauma events is illustrated: individuals with a history of traumatic childhood events were estimated to have lower follow-up BDI-II scores in CT relatively to IPT. This difference was significant for individuals with one or more traumatic childhood events as indicated by the Johnson–Neyman findings.

### The Personalized Advantage Index

#### PAI-indicated *v.* PAI non-indicated treatment

The selected variables were combined into the final regression model: AUC_7–24 months_ = *β*0 + (*β*1 × parental alcohol abuse) + (*β*2 × number of life events past year) + (*β*3 × number of childhood trauma events) + (*β*4 × number of life events past year × treatment) + (*β*3 × number of childhood trauma events × treatment). For each individual, long-term outcomes were predicted for CT and IPT using a fivefold cross-validation, and with these predictions, individual PAI scores were calculated. A total of 74 individuals had been assigned, by chance, to their PAI-indicated treatment, and 77 received, by chance, their PAI non-indicated treatment. Although the average follow-up BDI-II scores were lower for those who received their indicated treatment, this difference was not significant (indicated treatment = 14.5, non-indicated treatment = 17.2, *t* = 1.39, df = 149, *p* = 0.17). The effect size estimate (Cohen's *d*) of this difference was 0.23. Among those with the highest 60% PAI scores, 47 individuals received their PAI-indicated treatment and 44 individuals received their PAI non-indicated treatment. Mean follow-up BDI-II scores differed significantly between these groups (indicated treatment = 13.2, non-indicated treatment = 18.2, *t* = 2.22, df = 89, *p* = 0.03), with an effect size estimate of 0.47.

#### Individuals with a PAI indicating CT

**Fig. 3.**
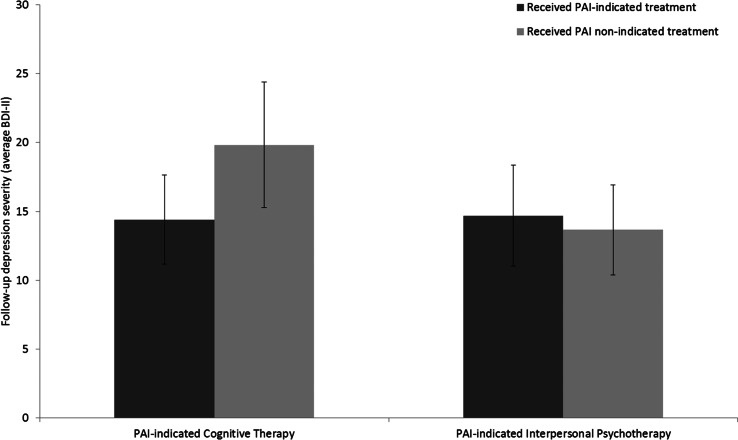
Comparison of the observed mean follow-up BDI-scores for individuals randomly assigned to their PAI-indicated optimal treatment *v.* their PAI-indicated non-optimal treatment. BDI-II, Beck Depression Inventory, Second Edition.

**Fig. 4.**
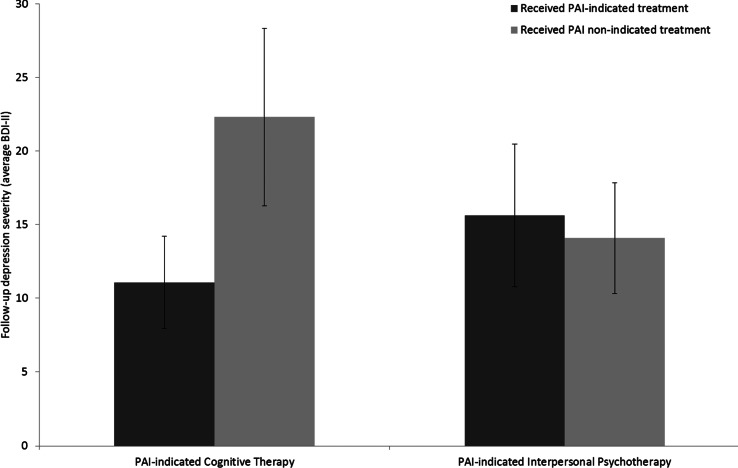
Subset of the sample with the top 60% PAI magnitude: comparison of the observed mean follow-up BDI-scores for individuals randomly assigned to their PAI-indicated optimal treatment *v.* their PAI-indicated non-optimal treatment. BDI-II, Beck Depression Inventory, Second Edition.

As shown in Fig. 3, for individuals whose PAI indicated CT as the optimal treatment, those who received CT (*n* = 43) reported lower follow-up BDI-II scores as compared to those who were allocated to IPT (*n* = 44; indicated treatment = 14.4, non-indicated treatment = 19.8, *t* = 1.95, df = 85, *p* = 0.05, Cohen's *d* = 0.42). As shown in Fig. 4, among the subset of individuals with a top 60% absolute value on the PAI, the difference in observed follow-up BDI-II scores was higher for those with a PAI-indicated CT, with lower follow-up depression severity for individuals randomized to CT (*n* = 25) as compared to those assigned to IPT (*n* = 22, indicated treatment = 11.1, non-indicated treatment = 22.3, *t* = 3.56, df = 45, *p* < 0.001, Cohen's *d* = 1.04).

#### Individuals with a PAI indicating IPT

As illustrated in Fig. 3, for those with a PAI indicating IPT, there was no significant difference in follow-up BDI-II scores between the individuals who were randomized to IPT (*n* = 31) *v.* CT (*n* = 33; indicated treatment = 14.7, non-indicated treatment = 13.7, *t* = 0.43, df = 62, *p* = 0.67, Cohen's *d* = −0.11). For the IPT-indicated individuals within the top 60% of PAI values, there was no significant difference between those receiving IPT (*n* = 22) *v.* those receiving CT (*n* = 22) (indicated treatment = 15.6, non-indicated treatment = 14.1, *t* = 0.52, df = 42, *p* = 0.61, Cohen's *d* = −0.16).

#### Long-term PAI *v.* post-treatment PAI

Long-term PAI scores were then compared to post-treatment PAI scores for each individual. The magnitude of the predictive advantage was not very consistent between long-term and post-treatment PAI scores, as indicated by a weak correlation (*corr.* = 0.33). Of the 76 individuals with a long-term PAI indicating CT, 46 (62.2%) had a post-treatment PAI indicating CT. Of the 58 individuals with a long-term PAI indicating IPT, 43 (74.1%) had a post-treatment PAI indicating IPT.

### Testing robustness of variable selection and model fitting

A secondary analysis was performed to examine the long-term PAI scores that would be obtained without ‘double-dipping’ during the variable selection stage (i.e. performing variable selection as well as weight setting in cross-validation folds, rather than performing variable selection in the full sample followed by weight setting in cross-validation folds). This analysis yielded results that were quite similar to the primary analysis. Mean follow-up BDI-II scores for individuals with a PAI-indicated treatment (*n* = 75) *v.* a PAI non-indicated treatment differed at the level of a non-significant trend (*n* = 76, indicated treatment = 14.0, non-indicated treatment = 17.8, *t* = 1.95, df = 149, *p* = 0.05) with an effect size of 0.32. Similar to the primary analysis, this difference was more pronounced among those with the highest 60% PAI scores [mean follow-up BDI-II scores indicated treatment (*n* = 46) = 13.7, non-indicated treatment (*n* = 45) = 19.9, *t* = 2.33, df = 89, *p* = 0.02], with an effect size of 0.49.

## Discussion

The aim of the current study was to replicate and extend the PAI method to long-term depression outcomes for CT and IPT for MDD. Using state-of-the-art variable selection techniques, one predictor (parental alcohol abuse) and two moderators (life events past year and childhood maltreatment) for long-term depression outcome following CT and IPT were identified. PAI scores were then computed for each individual based on the final model including the selected predictor and moderators using a cross-validation approach. PAI scores were evaluated by examining the observed follow-up depression severity scores, and by comparing the long-term PAI scores with the post-treatment PAI scores (Huibers *et al*., 2015). Overall, there was a small difference (2.7 points on the BDI-II) in observed depression severity for those assigned to their PAI-indicated treatment (lower follow-up depression severity) as compared to those assigned to their PAI non-indicated treatment (higher follow-up depression severity). As expected, this difference was more pronounced and statistically significant for individuals with a top 60% PAI score (5 points on the BDI-II). Notably, this difference was only present in individuals who were recommended to receive CT, whereas no mean differences were found for individuals recommended to receive IPT. Individual treatment recommendations and predicted advantages from the long-term PAI scores and the post-treatment were correlated, but only moderately.

### Predictors and moderators

In the current study, we identified parental alcohol abuse as a predictor, and recent life events and childhood maltreatment as moderators of long-term outcome. Parental alcohol abuse was associated with an unfavorable 17-month follow-up, irrespectively of the treatment received. This finding is in line with the research in adult children of alcoholics that reported an association between parental alcohol abuse and depressive mood (Kelley *et al*., 2010; Klostermann *et al*., 2011), and mood disorders (Cuijpers *et al*., 1999), although there is evidence that this association is mediated by adverse childhood experiences (Anda *et al*., 2002).

An increasing number of life events in the year before the start of therapy was associated with higher follow-up depression severity in IPT as compared to CT. This variable was also identified as one of the six moderators of the post-treatment PAI of the same study sample, with lower post-treatment depression severity in CT as compared to IPT (Huibers *et al*., 2015). In a previous study, a tendency was found for individuals with severe negative life events *prior to their onset of depression* to respond better to IPT than to CBT. However, findings of that same study indicated that response to treatment in individuals with severe negative life events *prior to their depression treatment* was superior in both CBT and IPT, relative to antidepressant medication (Bulmash *et al*., 2009).

The number of childhood trauma events was associated with an unfavorable 17-month follow-up in IPT relative to CT. Differential treatment outcomes for individuals with a history of childhood maltreatment have been described in previous studies (Nemeroff *et al*., 2003; Barbe *et al*., 2004; Asarnow *et al*., 2009; Lewis *et al*., 2010; Harkness *et al*., 2012). In line with the current findings, Harkness *et al*. (2012) reported lower response rates in IPT compared to CBT and antidepressant medication for individuals with childhood trauma. However, this differential effect did not sustain throughout a 12-month follow-up phase in that sample. In addition, previous studies comparing C(B)T to systemic behavioral family therapy, non-directive supportive therapy (Barbe *et al*., 2004) or antidepressant medication (Asarnow *et al*., 2009; Lewis *et al*., 2010) reported relatively poorer response rates in the C(B)T condition for adolescents with a history of childhood trauma.

In previous randomized trials comparing CT and IPT head-to-head, various predictors and moderators of post-treatment outcome were identified (Sotsky *et al*., 1991; Joyce *et al*., 2007; Luty *et al*., 2007; Ryder *et al*., 2010; Carter *et al*., 2011; Mulder *et al*., 2017). Only one study by Mulder *et al*. (2017) also identified predictors and moderators of long-term outcomes during maintenance CT and IPT following acute phase treatment. The findings of this study were not in line with our results: no significant moderators were identified, and personality variables were identified as significant predictors.

### Evaluating the long-term PAI

After the variable selection procedure, the three variables were combined in a final model and individual PAI scores were calculated. For those assigned to their PAI-indicated treatment, observed follow-up depression severity was non-significantly lower as compared to individuals randomized to their PAI non-indicated treatment. Similar to DeRubeis *et al*. (2014), for individuals that were estimated to have a relatively stronger need for a specific treatment (the top 60% PAIs), the observed depression severity scores of individuals receiving their PAI-indicated treatment were significantly lower than for those that received their PAI non-indicated treatment. The mean difference of this top 60% subset was 5 points on the BDI-II, which corresponds to a clinically meaningful difference (Hiroe *et al*., 2005). Interestingly, further analyses showed that this difference was primarily due to the outcomes observed in individuals whose PAI indicated CT. This finding can be understood by examining the relationships obtained with the individual variables in the final PAI model. As illustrated in Figs 3 and 4, each of the two moderators produced an ordinal pattern. One can interpret these moderator effects as follows: when an individual had two or more pre-treatment life events and/or one or more events of childhood maltreatment, CT would be indicated, whereas individuals with one or no life events and no childhood trauma have no indication of a meaningful difference between CT and IPT (Cohen and DeRubeis, 2018). These moderator effects and the differential performance of the PAI for CT *v.* IPT indicate a *specific* benefit of CT for a subgroup of individuals who suffered from childhood maltreatment events and recently experienced significant life events, whereas for the remainder of the individuals, no differential effect was observed. In clinical words, the advantage of CT over IPT only emerges among individuals with more complex life stories. Two possible explanations for these findings are that the more complex cases require a more active and structured type of therapy in which the therapist takes a more directive role, and the pivotal role of previous life experiences in the therapeutic procedure of cognitive restructuring of thoughts and schemas that lies at the heart of CT (whereas IPT, as practiced in this trial, only focused predominantly on the present).

### Long-term PAI *v.* post-treatment PAI comparison

The comparison between long-term PAI scores and post-treatment PAI scores (Huibers *et al*., 2015) indicated different treatment recommendations with different predicted advantages. Only the number of life events prior to treatment was a shared moderator. In addition, the final model of the post-treatment PAI included a higher number of predictors (gender, employment status, anxiety, personality disorder, and quality of life) and moderators (somatic complaints, cognitive problems, paranoid symptoms, interpersonal self-sacrificing, attributional style, and number of life events, Huibers *et al*., 2015) as compared to the model of the long-term PAI. There are several possible reasons for the lack of overlap between the post-treatment PAI and the long-term PAI. First, the post-treatment PAI and long-term PAI predicted two different types of outcomes: post-treatment depression severity *v.* an aggregated measure of follow-up depression severity. One could argue that these two outcomes represent two different phenomena with different combinations of moderators involved. Second, the time span between the pre-treatment variables and the predicted outcome is larger for the long-term PAI relatively to the post-treatment PAI. With this longer time period, relatively weaker variables lose their predictive power, resulting in fewer predictors and moderators for the long-term PAI. Third, for the variable selection procedure, different study samples were used for the long-term PAI (*n* = 151, intention to treat imputed dataset) and the post-treatment PAI (*n* = 134, only non-missing post-treatment BDI-II scores). Finally, different variable selection approaches were applied: a modified domain approach for the post-treatment PAI and a two-step machine learning approach for the long-term PAI. These different variable selection approaches reflect the heterogeneity of statistical approaches due to rapid developments in this area of research (Cohen and DeRubeis, 2018). In sum, the fact that the short- and long-term PAI advice did not overlap for each individual can be explained by a variety of reasons, and should not come as a surprise. Insofar as the inconsistency between short- and long-term indications are not an artifact but instead, reflect different influences on short- and long-term outcomes, this presents a problem that would need to be resolved if such work is to inform clinical practice. In other words, if different therapies are needed for optimal outcomes at different stages of MDD (i.e. post-treatment and the longer term) for the individual patient, this poses a real dilemma in the clinician's office when selecting a treatment.

### Limitations

The current study has limitations. First, the long-term PAI was not externally validated by applying it on an independent dataset. Although we used a cross-validation approach to compute the regression parameters of the final model, we used the full study sample for the variable selection procedure. To examine potential bias, we did a secondary analysis rerunning the complete process with fivefolds, producing five models that estimated the PAIs of individuals whose data were not used in any way to develop the algorithm that yielded the PAIs. This additional analysis produced very similar outcomes to those obtained in our primary analysis. Nevertheless, without external validation efforts, the degree to which this model can be generalized to new samples, populations, and treatment settings is yet unknown. Second, although we began our variable selection with 69 variables, it is still possible that relevant predictors or moderators were not included in our study. Third, individuals were allowed to seek additional treatment during follow-up. However, this did not significantly affect the long-term outcomes (Lemmens *et al*., 2019). Finally, our sample size of 151 individuals might be insufficient according to recent suggestions of sample size requirements for multivariate prediction models based on a single simulation study (Luedtke *et al*., 2019), although more research in this new area is needed to reach a final conclusion on this.

### Future directions

Despite these limitations, the current findings hold a promise for the PAI approach for longitudinal predictions for two treatments that are, on average, equally effective. Moving beyond post-treatment estimates, this type of PAI could guide treatment selection focusing on *keeping* a (formerly depressed) individual well over the long term. However, the long-term PAI is not ready for implementation. First of all, external validation in different populations with different treatment settings and time frames using prospective designs is needed. Second, a collaboration of different disciplinary lines to extend the number of potential predictors and moderators is of importance, combining biomarkers, dynamic assessments, clinical-rated, and self-report measures into one algorithm. Third, consideration of cost-effectiveness and feasibility of potential predictors and moderators should be a necessary part of new study designs (Kessler, 2018). Fourth, the use pooled datasets should be considered to have adequate power to develop multivariate prescriptive prediction models (Luedtke *et al*., 2019). Finally, methods that combine PAI predictions prior to treatment with updated predictions during treatment need to be studied further (e.g. Lutz *et al*., 2017). Ultimately, these efforts will hopefully lead to guided clinical decision-making, reducing the number of treatments needed to acquire and maintain remission.

## Supplementary material

For supplementary material accompanying this paper visit https://doi.org/10.1017/S0033291719003192. click here to view supplementary material
