# Supplementary material for: Precision medicine for long-term depression outcomes using the Personalized Advantage Index approach: cognitive therapy or interpersonal psychotherapy?
Source: Psychol Med. 2019 Nov 22;51(2):279–89. doi: 10.1017/S0033291719003192 (PMC7893512; doi:10.1017/S0033291719003192)
Supplement: Supplementary file 1 [file S0033291719003192sup001.docx]

**Supplemental Methods I: variable transformation**

**Table 1: Variable transformation**

| **DOMAIN I: Depression** | |  |
| --- | --- | --- |
| Recurrent episodes, n (%) | | Centered (no: -0.5, yes: 0.5) |
| Hopelessness, BHS, mean (S.D.) | | Standardized |
| **DOMAIN II: Demographics** | |  |
| Female, n (%) | | Centered (male: -0.5, female: 0.5) |
| Age, mean (S.D.) | | Standardized |
| Partner, n (%) | | Centered (yes: -0.5, no: 0.5) |
| Education level | | Centered |
| -  -  - | Low, n (%)  Intermediate, n (%)  High, n (%) | (low: -0.5,  (intermediate:0,  (high: 0.5) |
| Active employment, n (%) | | Centered (yes: -0.5, no: 0.5) |
| Treatment expectancy, mean (S.D.)  (0 = not successful - 10 = very successful) | | Standardized |
| **DOMAIN III: Psychological distress** | |  |
| General psychological distress, BSI | |  |
| -  -  -  -  -  -  - | Somatic complaints, mean (S.D.)  Cognitive problems, mean (S.D.)  Depression, mean (S.D.)  Anxiety, mean (S.D.)  Phobic Anxiety, mean (S.D.)  Hostility, mean (S.D.)  Paranoid Symptoms, mean (S.D.) | Standardized  Standardized  Standardized  Standardized  Standardized  Standardized  Standardized |
| Number of comorbid axis I disorders, SCID-I, mean (S.D.) | | Mean-centered |
| Number of comorbid axis II disorders, SCID-II, mean (S.D.) | | Mean-centered |
| Number of comorbid axis II traits, SCID-II, mean (S.D.) | | Mean-centered |
| **DOMAIN IV: General functioning** | |  |
| Social and work functioning, WSAS, mean (S.D.) | | Standardized |
| Level of impairment, RAND-36 | |  |
| -  -  -  -  - | Physical functioning, mean (S.D.)  Social functioning, mean (S.D.)  Role limitations (physical problems)  Role Limitations (emotional problems)  General health perception  Perceived health change during past year | Standardized  Standardized  Standardized  Standardized  Standardized  Standardized |
| **DOMAIN V: Psychological Processes** | |  |
| Dysfunctional Beliefs, DAS, mean (S.D.) | |  |
| -  - | Factor 1  Factor 2 | Standardized  Standardized |
| Interpersonal problems, IIP, mean (S.D.) | | Standardized |
| Self Liking and Self Competence, SLSC-R, mean (S.D.) | | Standardized |
| Rumination, RRS, mean (S.D.) | | Standardized |
| Attributional Style, ASQ, mean (S.D.) | | Standardized |
| **DOMAIN VI: Life and family history** | |  |
| Number of life events past year, mean (S.D.) | | Log transformation |
| Number of childhood trauma events, mean (S.D.) | | Mean-centered |
| Parental: one or both parents | |  |
| -  -  -  -  - | In treatment for illness, n (%)  With an anxiety disorder, n (%)  With depression, n (%)  With alcohol abuse, n (%)  With suicidality, n (%) | Centered (yes: -0.5, no: 0.5)  Centered (yes: -0.5, no: 0.5)  Centered (yes: -0.5, no: 0.5)  Centered (yes: -0.5, no: 0.5)  Centered (yes: -0.5, no: 0.5) |

BHS, Beck Hopelessness Scale; BSI, Brief Symptom Inventory; SCID-I, Structured Clinical Interview for DSM-IV Axis I disorders; SCID-II, Structured Clinical Interview for DSM-IV Axis II disorders; WSAS, Work and Social Adjustment Scale; DAS, Dysfunctional Attitudes Scale; IIP, Inventory of Interpersonal Problems; SLSC-R, Self Liking and Self Competence Scale Revised; RRS, Ruminative Response Scale; ASQ, Attributional Style Questionnaire
